# Comprehensive Review of Biological Functions and Therapeutic Potential of Perilla Seed Meal Proteins and Peptides

**DOI:** 10.3390/foods14010047

**Published:** 2024-12-27

**Authors:** Yangfan Hu, Huan Luo, Vasudeva Reddy Netala, He Li, Zhijun Zhang, Tianyu Hou

**Affiliations:** School of Chemical Engineering and Technology, North University of China, Taiyuan 030051, China; 13934877042@163.com (Y.H.); luohuan12142022@163.com (H.L.); vasunuc1922@gmail.com (V.R.N.); heli_science@nuc.edu.cn (H.L.); zjzhang@nuc.edu.cn (Z.Z.)

**Keywords:** perilla seed peptides, anti-aging, anti-fatigue, antidiabetic, anticancer, ACE inhibitory, intestinal health maintenance, immunomodulating activities

## Abstract

This comprehensive review explores the biological functions of *Perilla frutescens* seed proteins and peptides, highlighting their significant potential for health and therapeutic applications. This review delves into the mechanisms through which perilla peptides combat oxidative stress and protect cells from oxidative damage, encompassing free radical scavenging, metal chelating, in vivo antioxidant, and cytoprotective activities. Perilla peptides exhibit robust anti-aging properties by activating the Nrf2 pathway, enhancing cellular antioxidant capacity, and supporting skin health through the promotion of keratinocyte growth, maintenance of collagen integrity, and reduction in senescent cells. Additionally, they demonstrate antidiabetic activity by inhibiting α-amylase and α-glucosidase. The cardioprotective effects of perilla peptides are underscored by ACE-inhibitory activities and combat oxidative stress through enhanced antioxidant defenses. Further, perilla peptides contribute to improved gut health by enhancing beneficial gut flora and reinforcing intestinal barriers. In liver, kidney, and testicular health, they reduce oxidative stress and apoptotic damage while normalizing electrolyte levels and protecting against cyclophosphamide-induced reproductive and endocrine disruptions by restoring hormone synthesis. Promising anticancer potential is also demonstrated by perilla peptides through the inhibition of key cancer cell lines, alongside their anti-inflammatory and immunomodulating activities. Their anti-fatigue effects enhance exercise performance and muscle function, while perilla seed peptide nanoparticles show potential for targeted drug delivery. The diverse applications of perilla peptides support their potential as functional food additives and therapeutic agents.

## 1. Introduction

Perilla (*Perilla frutescens* L. Britton) is an annual herbaceous plant belonging to the Lamiaceae family, extensively cultivated across east Asia, including China, Japan, South Korea, and India [1]. Known for its various applications in traditional Chinese herbal medicine, perilla has gained prominence in culinary and medicinal contexts due to its unique aromatic essential oils and rich nutrient profile [2]. Perilla, commonly referred to as “deulkkae” in Korea, is widely utilized for its leaves, which serve as a spice, garnish, and food colorant. *Perilla frutescens* has a long history of cultivation exceeding two thousand years and is recognized as a medicine food homology plant [3]. The seeds of perilla are celebrated for their diverse physiological benefits, including anti-allergy, anti-inflammatory, antioxidant, anticancer, antibacterial, and neuroprotective effects [4,5,6]. The protein content in perilla seeds, which can reach up to 23.7%, is particularly notable for its richness in essential amino acids [5,6]. The seeds of perilla are valued for their high oil content, predominantly unsaturated fatty acids, including α-linolenic acid, which constitutes a significant portion of the seed’s fatty acid composition [7,8]. After oil extraction, the residual seed meal, known as perilla seed meal (PSM), remains a potentially rich source of bioactive compounds. PSM, with its higher protein content compared to unprocessed seeds, can be an important resource for producing functional peptides [9,10,11]. Despite its nutritional potential, PSM is often underutilized, frequently discarded, or used primarily as animal feed. This underuse of PSM presents an opportunity for innovation in utilizing this byproduct for producing valuable bioactive peptides, which could enhance its economic value and provide health benefits through functional food ingredients [12,13,14,15]. Peptides derived from PSM through enzymatic hydrolysis have shown promise in various biological activities, including antioxidant, antihypertensive, and antimicrobial properties [16,17,18,19,20,21,22]. The extraction of these peptides involves hydrolyzing PSM proteins using specific proteolytic enzymes under optimized conditions. Various enzymes, such as alcalase, Neutrase, trypsin, papain, and pepsin, each with distinct optimal pH, temperature, and activity conditions, are employed to break down proteins into bioactive peptides [17,18,19,20,21,22,23,24]. The process typically includes extracting peptides from PSM, purifying them using techniques like ultra-membrane filtration, Sephadex G-25 column chromatography, reverse-phase high-performance liquid chromatography (RP-HPLC), and automatic amino acid analyzer and mass spectrometry (LC-ESI-MS/MS) [25,26,27,28,29,30,31,32,33]. By harnessing the potential of PSM-derived peptides, researchers aim to enhance the value of this agricultural byproduct and contribute to the development of novel functional food ingredients with beneficial health effects.

In this review, we present an overview of the different biological properties of various perilla peptides, comprehensively explaining plausible mechanisms of action with schematic illustrations. Additionally, potential applications in health and disease prevention are discussed, highlighting the therapeutic promise of these bioactive compounds. Future perspectives on the development of peptide-based interventions and the need for further research are also addressed.

## 2. Biomedicinal Properties of Perilla Peptides

### 2.1. Free Radical Scavenging, Metal Chelating Ability, In Vivo Antioxidant, and Cytoprotective Effects of Perilla Peptides

Free radical scavenging, metal chelating ability, *in vivo* antioxidant, and cytoprotective effects are crucial aspects of understanding the protective mechanisms of various compounds against oxidative stress. Free radical scavenging involves neutralizing reactive oxygen species (ROS) that can cause cellular damage. Metal chelating ability refers to the capacity of a compound to bind and sequester metal ions, preventing them from catalyzing the formation of harmful free radicals [34]. *In vivo* antioxidant activity assesses the effectiveness of these compounds in reducing oxidative stress within living organisms. Cytoprotective effects highlight the ability of compounds to protect cells from damage caused by oxidative stress and other harmful conditions [35].

Yang et al., (2018) [16] isolated and purified two innovative peptides from perilla seed meal protein hydrolysate using alkaline protease. They characterized these as the dipeptides Phe-Tyr (FY) and Tyr-Leu (YL), with molecular masses of 328.3 and 294.3 Da, respectively. These dipeptides demonstrated potent *in vitro* antioxidant capabilities against DPPH, ABTS, and hydroxyl radicals, as well as a significant oxygen radical absorbance capacity. Moreover, they exhibited inhibitory effects on linoleic acid peroxidation and effectively mitigated lipid peroxidation in rat liver *in vivo.* Further analysis revealed that FY and YL could safeguard HepG-2 cells from hydrogen peroxide-induced oxidative damage without inducing cytotoxicity and also exhibited biocompatibility and cytoprotective effects on CHO cells [16]. Structure–activity relationship studies highlighted the indispensable role of the Tyr residue in the antioxidant activity of both FY and YL. This is attributed to the ability of Tyr to efficiently scavenge free radicals by donating a hydrogen atom from its phenolic group. These findings underscore the promising potential of protein hydrolysates and purified peptides from perilla seeds as functional food additives to enhance food quality. Additionally, they hold promise as ingredients in the medical and cosmetic industries for managing metabolic disorders stemming from elevated ROS levels [16,36,37]. Nevertheless, the *in vivo* antioxidant activity of these peptides and the mechanisms by which they protect against cellular oxidative stress warrant further investigation. Concurrently, the potential pro-apoptotic effects of high concentrations of FY on HepG-2 cells should be thoroughly explored.

In their research, Kim et al., (2019) [10] utilized trypsin hydrolysis to degrade proteins from PSM into antioxidant peptides. The resulting PSM protein hydrolysate was then refined through ultrafiltration and both preparative and analytical reverse-phase HPLC. Although the amino acid sequence of the purified antioxidant peptide (PAP1) in fraction IV remained elusive, the sequence of PAP2 in fraction V was identified as Ile-Ser-Pro-Arg-Ile-Leu-Ser-Tyr-Asn-Leu-Arg, possessing a molecular weight of 1330.77 Da. These two purified peptides exhibited potent antioxidant properties, manifesting considerable reducing power, as well as DPPH and ABTS radical scavenging activities [10]. Peptides ranging from 5 to 16 amino acids demonstrate superior antioxidant properties compared to larger polypeptides. This is attributed to their enhanced ability to traverse the intestinal barrier and more readily engage with free radicals [38,39]. The heightened antioxidant efficacy of these peptides is ascribed to their diminutive molecular weight (<3 kDa) and the presence of distinct amino acids, prominently Tyr, Leu, Ile, Pro, Ser, and Arg [40,41]. The phenolic hydroxyl group of tyrosine can donate hydrogen atoms to neutralize free radicals, forming a stable tyrosyl radical in the process, which minimizes further oxidative damage [16]. Leucine and isoleucine, being branched-chain amino acids, contribute to the hydrophobic interactions within the peptide structure, stabilizing it and reducing susceptibility to oxidative stress. Proline’s unique cyclic structure can disrupt secondary structures of proteins, enhancing the flexibility and accessibility of antioxidant sites within the peptides. Additionally, the hydroxyl group in serine can form hydrogen bonds and participate in radical scavenging, aiding in the stabilization and neutralization of free radicals [42,43]. Peptides with basic amino acids such as Arg exhibit excellent metal chelating ability [44]. These combined effects from different amino acids make the peptides potent antioxidants. These results emphasize the potential of PSM hydrolysates as promising reservoirs of natural antioxidants, underscoring their prospective utility across diverse industries.

In a study conducted by Henghui et al., (2023), a dodecapeptide was isolated and purified from PSM protein hydrolysate using alcalase enzyme extraction. The confirmed sequence of the peptide was Lys-Leu-Lys-Asp-Ser-Phe-Glu-Arg-Gln-Gly-Met-Val, with a molecular mass of 1437.8 Da [22]. This purified peptide exhibited notable antioxidant activity due to several key attributes. The aromatic amino acids phenylalanine and potentially tyrosine can donate electrons to stabilize free radicals, effectively neutralizing their detrimental effects [16]. Positively charged amino acids lysine and arginine act as free radical scavengers and can chelate metal ions [44]. These metal ions are catalysts in oxidative reactions and their chelation can inhibit the formation of ROS via Fenton and Haber–Weiss reactions. Additionally, the peptide has the potential to chelate transition metal ions, such as iron and copper, which are crucial in the generation of ROS through the Fenton and Haber–Weiss reactions. Polar amino acids, including serine, aspartic acid, glutamic acid, and glutamine can form hydrogen bonds with ROS and reactive nitrogen species (RNS), reducing their reactivity [42]. Hydrophobic amino acids leucine, methionine, and valine can interact with lipid radicals, stabilizing lipid membranes and shielding them from oxidative damage. Moreover, glycine, being the smallest amino acid, provides structural flexibility to the peptide, enabling effective interaction with various targets [45,46,47]. In summary, the antioxidant activity of the dodecapeptide can be attributed to its capacity to scavenge free radicals, chelate metal ions, stabilize lipid membranes, and form hydrogen bonds with reactive species. These properties are all facilitated by its distinctive amino acid sequence.

Hongyu et al., (2023) [48] extracted four peptide fractions (UF-1, UF-2, UF-3, and UF-4) from perilla seed meal using alkaline protease, and all these fractions showed free radical scavenging activity by inhibiting DPPH and ABTS free radicals. Among all the fractions, UF-4 showed effective DPPH and ABTS radical scavenging activity with IC_50_ values of 1.00 and 0.27 mg/mL. UF-4 contains 18 potential active peptides such as CFFYR, HFFWN, ACVFAFM, WFHYH, ILGFYW, WILPFVP, GALFL, HIFFH, GGGFRG, GLGAFL, FITFR, KAFFY, FIASFL, RIWRP, LAVFW, FLFNK, WVTFY, and GLFRI. These peptides protect cells from oxidative stress-induced damage by decreasing the levels of MDA, a marker of oxidative damage. The peptides protect cells from oxidative stress-induced damage by increasing intracellular levels of antioxidant factors like glutathione (GSH) and enhance the activity of antioxidant enzymes such as superoxide dismutase (SOD) and catalase (CAT). Further, the peptides reduce the activity of extracellular lactate dehydrogenase (LDH), indicating protective effects on cell membrane integrity during oxidative stress. They modulate pathways involved in cell death and survival and protect cells against apoptosis in response to oxidative stress [48].

In a study by Park and Yoon (2019), PSM protein hydrolysate (PPH) was produced using Flavourzyme, resulting in four distinct perilla peptide fractions with molecular masses ranging from less than 1 kDa to over 10 kDa. These fractions were assessed for their antioxidant activities using the ABTS, hydroxyl radical scavenging, and metal chelating ability assays. The smallest peptide fraction, <1 kDa, demonstrated the most potent antioxidant activity against ABTS, followed by the 1–3 kDa, <3–5 kDa, and 5–10 kDa fractions, and the original PPH. Conversely, the >10 kDa fraction exhibited the least activity. For hydroxyl radical scavenging, the <1 kDa fraction was the most effective, followed by the 1–3 kDa, 3–5 kDa, and 5–10 kDa fractions, and PPH, while the >10 kDa fraction showed the lowest activity. In terms of metal chelating ability, PPH displayed the most significant chelating capacity, followed by the 1–3 kDa, <1 kDa, 3–5 kDa, and >10 kDa fractions, with the 5–10 kDa fraction showing the weakest Fe^2+^ chelating ability. Given these findings, it is hypothesized that the 1–3 kDa fraction contains a higher proportion of hydrophobic amino acids compared to <1 kDa fraction [14]. Peptides with lower molecular weights generally exhibited superior antioxidant activity, with the exception of Fe^2+^ chelating ability, compared to peptides with higher molecular weights. Generally, the capacity of peptides to act as electron donors and to react with free radicals tends to increase with decreasing molecular weight, making them more effective in halting chain reactions [49]. The <1 kDa fraction from PPH displayed notable antioxidant activity, suggesting its potential as an effective antioxidant for mitigating or preventing damage caused by free radicals in both food and biological systems.

Kim and Yoon (2020) evaluated the antioxidant capacities of the PSM protein hydrolysates using DPPH and ABTS radical scavenging assays [50]. The DPPH radical scavenging activities of the PSM protein hydrolysates were ranked in the following order: Neutrase exhibited the most potent activity, followed by trypsin, papain, pepsin, and alcalase. The IC_50_ values for the DPPH radical scavenging activity ranged from the lowest in Neutrase to the highest in alcalase. The superior DPPH radical scavenging activity of the Neutrase-treated hydrolysate could be attributed to the high content of glutamic acid, which has strong antioxidant properties due to its carboxyl and amino groups capable of chelating metal ions. Trypsin-treated hydrolysate demonstrated better DPPH radical scavenging activity compared to the hydrolysates treated with papain, pepsin, and alcalase. This can be explained by the amino acid composition, as negatively charged amino acids like aspartic acid and glutamic acid exhibit greater digestive stability than positively charged amino acids such as arginine, histidine, and lysine. Consequently, the Neutrase-treated hydrolysate, rich in negatively charged amino acids, could maintain its DPPH radical scavenging activity under digestive conditions. The ABTS radical scavenging assay evaluates the antioxidant capacity of molecules, both lipophilic and hydrophilic, based on their ability to donate electrons or hydrogen. The enzyme used for hydrolysis significantly influenced the ABTS radical scavenging activity of the hydrolysates, likely due to enzyme specificity. Trypsin-treated hydrolysates demonstrated the highest ABTS radical scavenging capacity, followed by alcalase, Neutrase, papain, and pepsin. The superior ABTS radical scavenging activity of the trypsin-treated hydrolysate may be attributed to the presence of hydrophobic amino acids that react with free radicals, stabilizing them and terminating the radical chain reaction. Additionally, the electrophoretic pattern of the trypsin hydrolysate revealed more bands with molecular weights below 14.2 kDa. Nguyen et al., (2017) reported that protein hydrolysates with molecular weights below 13 kDa could exhibit antioxidant activity. Furthermore, low molecular weight peptides are generally more stable during gastrointestinal digestion compared to high molecular weight peptides. Thus, the trypsin-treated hydrolysate is expected to maintain its structural stability during simulated gastrointestinal digestion [51].

**Figure 1:**
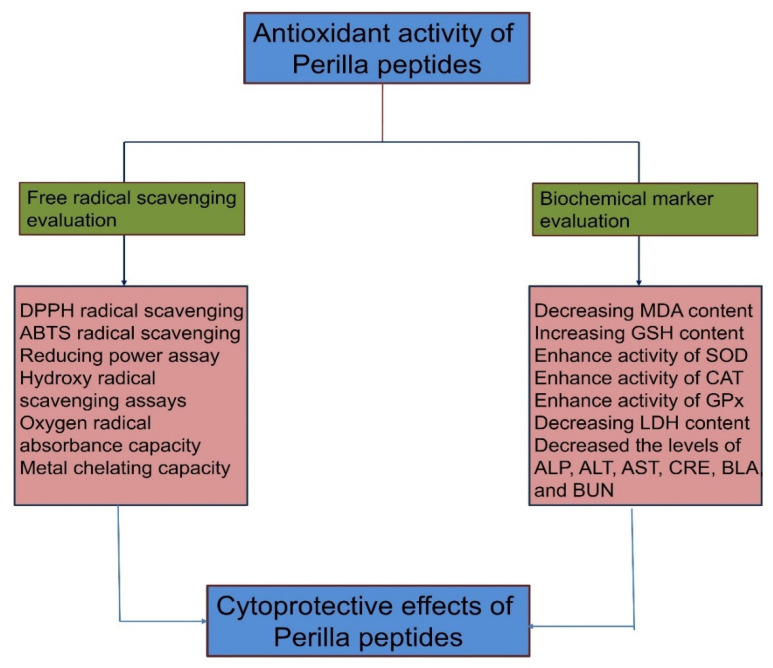
Represents antioxidant activity of perilla peptides leads cytoprotective effect through metal chelation, free radical scavenging, and in vivo assays. (Figure created using Biorender.com).

The antioxidant activity of perilla peptides leads to cytoprotective effects against oxidative damage through multiple pathways (Figure 1). They achieve this by effectively scavenging free radicals, as evidenced by assays like DPPH, ABTS, and oxygen radical absorbance capacity. Additionally, they demonstrate strong metal chelating capacity, which reduces metal-catalyzed oxidative reactions. Perilla peptides also enhance the activity of key antioxidant enzymes, such as SOD, CAT, and glutathione peroxidase (GSH-Px), thereby boosting the cellular defense system. Furthermore, they decrease malondialdehyde (MDA) content, a marker of lipid peroxidation, while increasing GSH levels, which protect against cellular damage. These peptides also lower levels of biochemical markers like LDH, alkaline phosphatase (ALP), alanine aminotransferase (ALT), aspartate aminotransferase (AST), creatinine, blood lactate (BLA), and blood urea nitrogen (BUN), indicating reduced tissue injury and improved cellular health. Perilla peptides are emerging as potent antioxidant agents, further leading to various other beneficial biological functions.

### 2.2. Anti-Aging Properties of Perilla Peptides

In recent years, there has been growing interest in the use of natural bioactive compounds for anti-aging applications. Among these, peptides derived from natural sources have shown significant promise due to their potent antioxidant and anti-senescent properties [52]. *P. frutescens*, a traditional medicinal plant, has been recognized for its various health benefits, including its potential in skin care. In a recent study by Wang et al., (2024) [24], perilla seed protein hydrolysate was prepared using neutral protease and subsequently fractionated via ultrafiltration into three distinct fractions based on molecular weight: F1 (MW < 3 kDa), F2 (3 kDa < MW < 5 kDa), and F3 (MW > 5 kDa). Perilla seed protein hydrolysate F2 fraction, containing peptides with molecular weights less than 5 kDa, demonstrated significant antioxidant activity [24]. The safety profile of F2 was evaluated using HaCaT (human keratinocyte) cells, where it exhibited no cytotoxic effects at concentrations up to 400 μg/mL, underscoring its potential for safe application in skin-related treatments. F2’s protective effect against oxidative stress was evident as it significantly reduced reactive oxygen species (ROS) levels in H_2_O_2_-treated HaCaT cells. This indicates its efficacy in mitigating oxidative damage, a crucial factor in skin aging and other oxidative stress-related conditions. Additionally, the anti-senescent activity of F2 was reported in HFF-1 (human foreskin fibroblast) cells using a D-galactose-induced aging model. F2 markedly reduced the number of senescent cells, suggesting its potential as an anti-aging agent. These findings highlight the dual functionality of F2 in both antioxidant defense and anti-aging processes, making it a promising candidate for further development. The study employed LC-MS/MS to identify 1696 peptide sequences from the F2 fraction. Nine peptides (RAW, FGRL, NFF, PMR, WGRP, MYF, FAGR, WFL, and GEMF) were predicted to possess strong antioxidant properties based on in silico analysis. These peptides’ interactions with the Keap1 protein, a critical sensor for oxidative stress and electrophiles, were explored through molecular docking. The strong binding affinities of these peptides to Keap1 suggest their potential to activate the Nrf2 pathway, a key regulator of antioxidant defense mechanisms. Keap1 plays a pivotal role in the Keap1-Nrf2 signaling pathway, which is essential for cellular defense against oxidative stress. Under normal conditions, Keap1 promotes the degradation of Nrf2, maintaining low basal levels of Nrf2. However, under oxidative stress, Keap1 undergoes modifications that lead to the stabilization and nuclear translocation of Nrf2 [53]. Nrf2 then induces the expression of antioxidant and cytoprotective genes, thus enhancing the cell’s ability to counteract oxidative damage. By interacting with Keap1, the identified peptides may disrupt its inhibitory interaction with Nrf2, thereby facilitating the activation of the Nrf2 pathway and enhancing cellular antioxidant capacity [54]. The bioactivity of the peptides was further confirmed, with seven out of the nine peptides promoting the proliferation of HaCaT cells. Notably, NFF, WGRP, MYF, FAGR, and PMR exhibited the most significant effects. These peptides were also tested for their antioxidant properties, where NFF and PMR stood out, particularly in reducing ROS levels in HaCaT cells exposed to H_2_O_2_. NFF and PMR also demonstrated notable anti-aging properties in D-gal-induced senescent HFF-1 cells by significantly reducing the number of senescent cells and the activity of β-galactosidase. These peptides modulated the expression of matrix metalloproteinases (MMPs) and collagen genes, reducing MMP expression while increasing COL-I and COL-III expression. This suggests their role in maintaining skin integrity and preventing the breakdown of collagen, which is crucial for skin aging.

**Figure 2:**
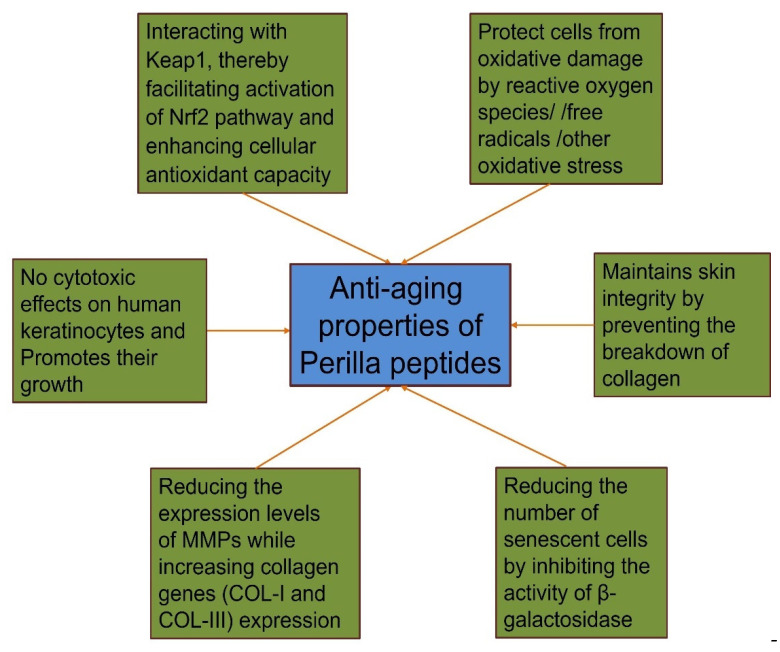
Schematic representation of anti-aging properties of perilla peptides via multiple mechanisms. Figure created using Biorender.com.

Perilla peptides exhibit robust anti-aging properties through multiple pathways. They interact with Keap1 to activate the Nrf2 pathway, enhancing cellular antioxidant capacity and protecting cells from oxidative damage. The peptides show no cytotoxic effects on human keratinocytes and promote their growth, supporting skin health. By maintaining skin integrity, they prevent collagen breakdown, reduce MMP expression, and increase collagen genes (COL-I and COL-III) expression. Additionally, they decrease the number of senescent cells by inhibiting β-galactosidase activity. These combined mechanisms make perilla peptides a promising candidate for anti-aging treatments (Figure 2).

### 2.3. Antidiabetic Activity of Perilla Peptides

The inhibition of α-amylase and α-glucosidase is crucial for controlling the rise in blood glucose levels, as these enzymes play pivotal roles in the digestion of carbohydrates and the subsequent elevation of blood glucose [55]. α-Amylase catalyzes the breakdown of starch into simpler sugars, while α-glucosidase further hydrolyzes these sugars into glucose, which is then absorbed into the bloodstream. By inhibiting these enzymes, it is possible to slow down carbohydrate digestion and glucose absorption, thereby reducing postprandial blood glucose spikes. This mechanism is particularly beneficial for managing non-insulin-dependent diabetes mellitus (NIDDM), obesity, and hyperglycemia, conditions where the regulation of blood glucose is compromised [55,56]. The α-amylase inhibitory activity of all peptide fractions and perilla peptide hydrolysate (PPH) ranged from 727.89 to 757.18 µg/mL [14]. Compared to peptides from other sources, perilla peptides demonstrated IC_50_ values 1–10 times lower, indicating their superior α-amylase inhibitory properties [57]. In terms of α-glucosidase inhibitory activity, the <1 kDa fraction exhibited the highest potency with an IC50 value of 54.51 µg/mL, while the >10 kDa fraction showed the least activity with an IC50 value of 1119.00 µg/mL. The ranking of α-glucosidase inhibitory activity among the peptides and PPH is as follows: <1 kDa, 3–5 kDa, 1–3 kDa, PPH, 5–10 kDa, and >10 kDa. These findings suggest that peptides derived from PPH could serve as potential therapeutic agents for managing postprandial hyperglycemia. The superior α-amylase inhibitory activity of perilla peptides, indicated by their low IC50 values, highlights their potential effectiveness in reducing the initial breakdown of starch into sugars, thus modulating the early stages of carbohydrate digestion. The particularly strong α-glucosidase inhibitory activity of the <1 kDa fraction further underscores its promise as a therapeutic agent, as it can significantly impede the final step of glucose production and absorption in the small intestine. The effectiveness of perilla peptides in inhibiting both α-amylase and α-glucosidase positions them as multifunctional agents capable of providing comprehensive glucose regulation. This dual inhibition approach is advantageous in providing a more sustained and controlled reduction in blood glucose levels, compared to targeting a single enzyme. Additionally, the smaller molecular weight peptides (<1 kDa) appear to be more effective, which may be due to their ability to interact more readily with the enzyme active sites, thus providing stronger inhibitory effects. Given their potent enzyme inhibitory activities, perilla peptides could be further developed into functional food ingredients or supplements aimed at managing diabetes and related metabolic disorders. Their natural origin and dual inhibitory properties offer a promising alternative to synthetic inhibitors, which often come with undesirable side effects. Future research could focus on optimizing the extraction and purification processes to maximize the yield of the most effective peptide fractions, as well as conducting clinical trials to validate their efficacy and safety in human subjects.

### 2.4. Antihypertensive Potential and Cardioprotective Effects of Perilla Peptides

**Figure 3:**
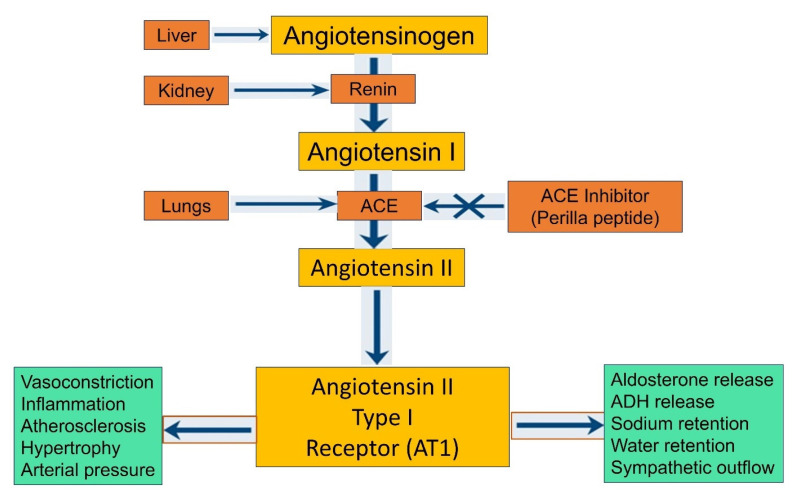
Schematic representation ACE-inhibitory activity of perilla peptides leads to cardioprotective effects. Figure created using Biorender.com.

The inhibition of angiotensin I-converting enzyme (ACE) serves as a crucial indicator for evaluating the antihypertensive properties of biological components. ACE is a central enzyme in the renin–angiotensin system (RAS) that regulates blood pressure and fluid balance in the body [58]. By converting angiotensin I to the potent vasoconstrictor angiotensin II, ACE directly influences vascular resistance and blood pressure. Therefore, inhibiting ACE can effectively lower blood pressure by preventing the formation of angiotensin II, reducing vasoconstriction, and promoting vasodilation. This mechanism is the basis for many antihypertensive drugs, highlighting the therapeutic significance of ACE inhibitors (Figure 3). ACE inhibitory peptides derived from natural sources are particularly promising, as they may offer effective and safer therapeutic options with fewer side effects compared to conventional ACE inhibitors [59,60]. Park and Yoon et al., (2019) [14,20] prepared various peptide fractions and protein hydrolysates from PSM using Flavourzyme at pH 7.0 at 50 °C for 4 h and collected various fractions as <1, 1–3, 3–5, 5–10, and >10 kDa fractions. Among the peptide fractions isolated from perilla, the <1 kDa fraction demonstrated the most robust ACE inhibitory activity, while the 5–10 kDa fraction exhibited the least activity. This observation aligns with existing research, which consistently shows that low molecular weight peptides possess more potent ACE-inhibitory activity compared to high molecular weight peptides. Notably, the peptide hydrolysate (PPH), >10 kDa, and 1–3 kDa fractions displayed comparable inhibitory activities, with no significant differences observed among these fractions [14]. In addition to ACE inhibition, these peptides exhibited significant antioxidant activity. This dual functionality further enhances their therapeutic potential. Antioxidants are vital in neutralizing free radicals and reducing oxidative stress, which is implicated in various cardiovascular diseases. Therefore, peptides that can inhibit ACE and exhibit antioxidant properties offer a multifaceted approach to cardiovascular health. Given their dual capabilities in both ACE inhibition and antioxidant activity, perilla-derived peptides hold promise not only as antihypertensive agents but also as beneficial components for overall cardiovascular health. The <1 kDa fraction, in particular, which displayed significant ACE-inhibitory and antioxidant activities, could be considered a particularly promising candidate for further development as a multifunctional therapeutic agent for hypertension and oxidative stress-related conditions. The findings that low molecular weight peptides exhibit stronger ACE-inhibitory activity can be attributed to their ability to interact more effectively with the ACE active site [61,62]. Smaller peptides can fit more easily into the enzyme’s binding pockets, leading to better inhibition. This principle underpins the development of peptide-based ACE inhibitors, which aim to mimic these interactions to achieve therapeutic effects [61,62,63]. The additional antioxidant properties of these peptides further their potential clinical utility. Oxidative stress is a major contributing factor to the development and progression of hypertension and other cardiovascular diseases. By mitigating oxidative stress, these peptides could help protect against endothelial dysfunction, a key event in the pathogenesis of hypertension and atherosclerosis [64,65]. Moreover, the combination of ACE inhibition and antioxidant activity suggests that perilla peptides could provide comprehensive cardiovascular protection. They could lower blood pressure, reduce oxidative damage, and improve endothelial function, thereby addressing multiple aspects of cardiovascular health.

The ACE-inhibitory activities of PSM protein hydrolysates were assessed, revealing IC50 values ranging from 3.76 to 5.2 mg/mL. Among them, the hydrolysates produced using pepsin and trypsin exhibited the most potent ACE-inhibitory activity. In contrast, the hydrolysates from papain, alcalase, and Neutrase displayed moderate ACE-inhibitory effects [9]. The pepsin-mediated hydrolysis is particularly noteworthy for generating biologically active ACE-inhibitory peptides. Pepsin exhibits broad specificity for peptide bonds and acts primarily on the N-terminus of aromatic amino acids [66]. This enzyme can hydrolyze a wide array of proteins to produce hydrolysates that retain ACE-inhibitory activity. The superior ACE-inhibitory activity of the pepsin-derived hydrolysate may be attributed to its high glycine content [67]. It has been observed that the amino acid composition can significantly influence the gastrointestinal digestive stability of the hydrolysates. Previous studies have indicated that peptides rich in acidic amino acids exhibit greater stability in the gastrointestinal tract compared to peptides containing predominantly neutral and basic amino acids. Given that the pepsin-derived hydrolysate demonstrated a high content of acidic amino acids, this finding correlates with its enhanced ACE-inhibitory activity and increased gastrointestinal stability [68]. This study underscores the potential of PSM protein hydrolysates, especially those derived from pepsin, as promising sources of ACE-inhibitory peptides. These peptides could be valuable in the development of functional foods and nutraceuticals aimed at managing hypertension and promoting cardiovascular health. Their gastrointestinal stability further enhances their potential as bioactive compounds with therapeutic benefits. As the search for natural and safe alternatives to synthetic chemicals continues, the exploration of natural peptides and bioactive compounds with ACE-inhibitory activity offers a promising avenue for the development of effective and safer therapeutic interventions for hypertension and cardiovascular diseases. Further research is warranted to explore the full therapeutic potential, mechanisms of action, and safety profiles of these ACE-inhibitory peptides derived from natural sources.

### 2.5. Perilla Peptides for Intestinal Health Maintenance

**Figure 4:**
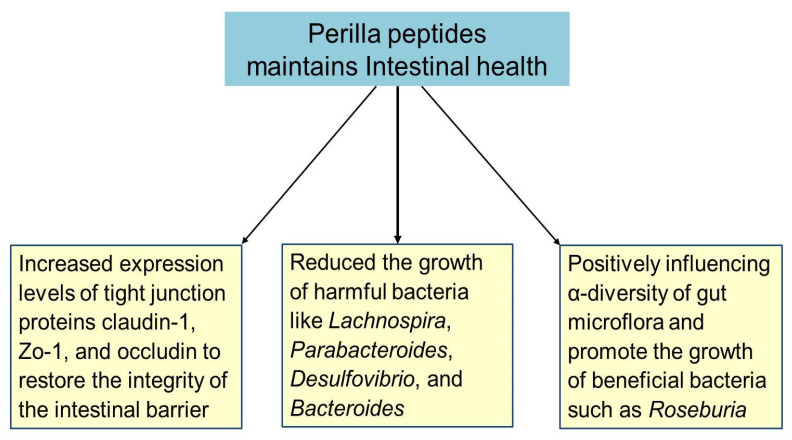
Perilla peptide maintains intestinal health through multiple pathways. Figure created using Biorender.com.

The gut harbors a vast array of microorganisms that significantly impact host nutrition, physiology, immunity, and metabolism [69]. Alterations in these gut microbes and impaired gut barrier function are closely linked to the progression of metabolic diseases. In patients with metabolic diseases, the number and nature of gut microorganisms are often disrupted, and bacterial toxins can exacerbate the disease and its complications [70]. One key indicator of intestinal health is the α-diversity of gut flora, reflecting the stability and robustness of the intestinal environment [71]. Perilla peptides have demonstrated a crucial role in maintaining intestinal health by positively influencing gut microflora and strengthening the gut barrier. They significantly improve the diversity and composition of gut microorganisms, leading to an increase in the α-diversity of the intestinal flora. This enhancement indicates a more stable and healthier gut environment, thereby improving intestinal microecology. Specifically, perilla peptides promote the growth of beneficial bacteria such as *Roseburia*. *Roseburia* produces butyrate, a short-chain fatty acid that regulates inflammation, repairs the intestinal epithelial barrier, and protects kidney function [31,72]. Conversely, harmful bacteria like *Lachnospira*, *Parabacteroides*, *Desulfovibrio*, and *Bacteroides*, which are commonly increased in patients with chronic metabolic diseases, are significantly reduced by perilla peptides. *Desulfovibrio*, for example, is a pathogen that produces toxic sulfides, disrupts the intestinal barrier, and is linked to lipid metabolism disorders. Similarly, *Parabacteroides* and *Lachnospiraceae* are associated with liver damage and the development of obesity and diabetes, respectively. Perilla peptides effectively decrease the abundance of these harmful bacteria, showcasing their regulatory effect on intestinal microorganisms [31,73]. The intestinal epithelium relies on tight junction proteins, such as claudin-1, Zo-1, and occludin, to maintain its barrier function against toxins and pathogens [74]. In conditions like adenine-induced liver and kidney injuries, high ammonia levels disrupt these junctions, leading to increased intestinal permeability and systemic inflammation. Perilla peptides have been shown to increase the expression of claudin-1, Zo-1, and occludin, thereby restoring the integrity of the intestinal barrier and reducing serum endotoxin levels. This restoration is critical for preventing the infiltration of harmful metabolites and reducing pro-inflammatory factors secreted by immune cells [31]. The beneficial effects of perilla peptides can be attributed to their rich amino acid composition, including Asp, Leu, Thr, Tyr, Ser, Phe, Glu, His, Gly, Lys, Ala, Arg, Val, Pro, Met, Cys, and Ile, with an average molecular mass of 760 kDa. These amino acids play vital roles in protein synthesis, cellular repair, and metabolic processes. For instance, Glu and Arg are crucial for maintaining the gut barrier function and supporting immune responses [75,76]. Pro and Gly contribute to collagen synthesis, which is essential for the structural integrity of the intestinal lining [77,78]. These amino acids collectively enhance the gut’s ability to repair and maintain itself, supporting overall intestinal health [75,76,77,78]. Thus, perilla peptides contribute significantly to intestinal health by improving gut microflora diversity, reducing harmful bacteria, enhancing the expression of tight junction proteins, and restoring the intestinal barrier (Figure 4). Their rich amino acid composition further supports these beneficial effects, making perilla peptides a potent natural intervention for maintaining and improving gut health.

### 2.6. Nitric Oxide (NO) Inhibitory Activity and Anti-Inflammatory Potential of Perilla Peptides

NO plays a pivotal role in various physiological processes, including neurotransmission and vasodilation, and is also implicated in the pathogenesis of inflammatory diseases. Excessive production of NO can lead to tissue damage and chronic inflammation. Therefore, the inhibition of NO production is crucial for the prevention and management of inflammatory conditions [79]. Natural peptides and bioactive compounds with NO inhibitory activity have gained attention as potential anti-inflammatory agents due to their perceived safety profile and fewer side effects compared to synthetic chemicals. The NO inhibitory activities of five PSM protein hydrolysates were reported using LPS-stimulated RAW 264.7 macrophages, with a lower IC_50_ value indicating higher NO inhibitory activity. The IC_50_ values for the treated hydrolysates were as follows: alcalase (3 mg/mL), Neutrase (2.99 mg/mL), trypsin (2.67 mg/mL), papain (2.98 mg/mL), and pepsin (2.11 mg/mL). Remarkably, the pepsin-treated hydrolysate exhibited the most potent NO inhibitory activity, whereas the trypsin-treated hydrolysate demonstrated the least. The PSM hydrolysates prepared using alcalase, Neutrase, and papain also showed equally effective NO inhibitory activity [50]. Pepsin, an endopeptidase, can cleave peptide bonds of N-terminal amino acids within proteins without converting the peptides into monomers. Several studies have highlighted the utility of pepsin hydrolysis for producing biologically active hydrolysates [80]. In the study, the pepsin-treated hydrolysate from PSM displayed the highest NO inhibitory activity among all the proteolytic hydrolysates examined.

Adenine-treated mice exhibited increased levels of the inflammatory factors IL-1β, IL-6, and TNF-α in the serum, indicating a pronounced inflammatory response. However, following perilla peptide administration, the levels of these inflammatory factors decreased to varying degrees. Notably, the reductions in IL-1β and IL-6 were particularly significant after perilla peptide treatment. Western blot analysis further showed a marked increase in the expression of IL-1β, IL-6, and TNF-α proteins in the kidneys of the adenine-treated mice. After perilla peptide administration, the expression of these proteins was notably reduced, with perilla peptides leading to a substantial decrease in the expression of all three proteins [31].

Hongyu et al., (2023) [48] extracted four peptide fractions (UF-1, UF-2, UF-3, and UF-4) from PSM using alkaline protease. Among all the fractions, UF-4 contains 18 potential active peptides such as CFFYR, HFFWN, ACVFAFM, WFHYH, ILGFYW, WILPFVP, GALFL, HIFFH, GGGFRG, GLGAFL, FITFR, KAFFY, FIASFL, RIWRP, LAVFW, FLFNK, WVTFY, and GLFRI. These peptides protect cells from oxidative stress-induced damage by decreasing the levels of MDA, increasing the intracellular levels of GSH; enhance the activity of antioxidant enzymes such as SOD and CAT; and reduce the activity of extracellular LDH. They modulate pathways involved in cell death and survival and protect against apoptosis in response to oxidative stress, modulate inflammatory pathways to reduce oxidative stress-induced inflammation, and inhibit key inflammatory signals such as the TNF and IL-17 pathways [48]. The findings underscore the potential of perilla peptides in attenuating the inflammatory response in mice

Anti-inflammatory activity is a critical property of bioactive compounds, as chronic inflammation is a key contributor to various diseases, including cardiovascular diseases, cancer, and neurodegenerative disorders [81]. The development of natural peptides and bioactive compounds with anti-inflammatory properties is of great interest due to their potential therapeutic benefits and reduced side effects compared to synthetic anti-inflammatory agents [82]. This study highlights the potential of PSM protein hydrolysates, particularly those treated with pepsin, as valuable sources of natural peptides with potent NO-inhibitory and anti-inflammatory activities. These peptides could serve as promising candidates for the development of functional foods and nutraceuticals aimed at managing inflammatory conditions and promoting overall health [48,50].

### 2.7. Immunomodulatory Effects of Perilla Peptides

IL-2 and IFN-γ are pivotal immune regulatory factors in the body, playing crucial roles in modulating both innate and adaptive immune responses. IL-2 is primarily produced by activated T cells and is essential for the proliferation and differentiation of T cells, while IFN-γ is a key cytokine produced by activated T cells and NK cells, playing a critical role in antiviral and antitumor immune responses. IgG is the predominant immunoglobulin in serum, responsible for mediating the humoral immune response, including neutralization of pathogens and activation of the complement system. In rats with cyclophosphamide-induced gonadotoxicity, the serum concentrations of IL-2, IFN-γ, and IgG were found to be significantly reduced, indicating compromised immune function. However, promisingly, oral administration of perilla seed protein and peptide led to increased levels of IL-2, IFN-γ, and IgG in the serum, thereby enhancing the immune response and restoring immune homeostasis. This immunomodulatory effect of Perilla seed protein and peptide may be attributed to their rich content of specific amino acids, such as Thr, Glu, Gly, Leu, and Arg. These amino acids have been reported to participate in immunoglobulin synthesis and immune regulation, thereby enhancing the production of IL-2, IFN-γ, and IgG. The restoration of these key immune factors underscores the potential of perilla seed protein and peptide as effective immunomodulatory agents for mitigating the immune disturbances associated with cyclophosphamide-induced gonadotoxicity and improving overall immune function. Thus, the significant increase in the serum levels of IL-2, IFN-γ, and IgG following perilla seed protein and peptide administration highlights their potential as promising therapeutic interventions for enhancing immune function and restoring immune balance in conditions of cyclophosphamide-induced gonadotoxicity. The immunopharmacological effects of perilla peptides emphasize their potential utility in supporting immune health and combating the adverse effects on immune function, thereby contributing to overall well-being and quality of life.

### 2.8. Perilla Peptides: Hepatoprotective, Renal Protective, and Testicular Protective Effects

In a study conducted by Miangliang et al., (2021) [31], PSM hydrolysate was prepared using neutral protease and papain enzymes. From this PSM hydrolysate, perilla peptides were isolated with an average molecular mass of 760 Da and amino acid composition that included Asp, Thr, Ser, Glu, Gly, Ala, Val, Met, and Ile. Mingliang et al., (2021) [31] demonstrated that cyclophosphamide-induced sexual impairment in rats led to organ atrophy of the spleen, thymus, kidneys, and testes. However, the administration of perilla seed peptides reversed this atrophy and significantly increased the organ indexes. Both low-dose and high-dose perilla seed peptide treatments markedly enhanced the spleen and thymus indexes, indicating substantial immune function improvement. Additionally, the testis index saw a notable rise, suggesting that perilla peptides contribute significantly to reproductive health recovery. While the kidney index remained stable across all groups, the comprehensive data unequivocally suggest that perilla seed peptides play a crucial role in reversing organ atrophy and enhancing organ function, thereby offering a potent therapeutic option for addressing sexual dysfunction-related organ impairment [83]. In adenine-induced injury models of mice, the kidney and liver indices were significantly decreased compared to the control. However, treatment with various doses of perilla peptides reversed this decline, suggesting a potential role for perilla peptides in restoring the damaged organ conditions, particularly for the kidney and liver [31]. These findings highlight the potential beneficial effects of perilla peptides in mitigating adenine-induced organ damage and promoting organ health in experimental mouse models.

In the adenine-induced injury model group, mice exhibited elevated serum levels of liver enzymes and kidney markers, including ALP, AST, ALT, BUN, uric acid, and creatinine, indicating potential liver and kidney damage or dysfunction. However, upon intervention with perilla peptides, the serum levels of these enzymes and markers were effectively reduced compared to the model group. In the kidneys of the mice treated with adenine, the activities of SOD, CAT, and GSH-Px were markedly reduced, along with a decrease in GSH content, compared to the normal group. Conversely, the MDA levels significantly increased, indicating severe oxidative stress. After the administration of perilla peptides, these parameters improved to varying degrees. In the livers of the model group mice, adenine exposure led to decreased activities of SOD, CAT, and GSH-Px, as well as a reduction in GSH levels and an increase in MDA levels. However, these changes were mitigated to varying extents following the perilla peptide treatment. In the testes of the mice, perilla peptide intervention resulted in elevated activities of SOD, CAT, and GSH-Px; an increase in GSH content; and a decrease in MDA content. Perilla peptides effectively enhanced the activities of SOD, CAT, and GSH-Px and increased GSH content across the kidneys, livers, and testes. Concurrently, they reduced MDA levels in these organs [31,83].

Perilla peptides demonstrated an ameliorative effect on rats with cyclophosphamide-induced impaired sexual function. Rats with impaired sexual function exhibited decreased SOD activity and increased MDA content in the testes and kidneys, indicative of oxidative stress and cellular damage. Treatment with perilla peptides effectively reversed these effects, showing increased SOD activity and decreased levels of MDA content in the testes and kidneys, thereby alleviating oxidative damage and improving organ function [31,83]. The observed restoration of SOD activity and reduction in MDA content further highlight the potential therapeutic benefits of perilla peptides in mitigating the adverse effects of cyclophosphamide-induced toxicity and oxidative stress. Perilla peptides enhance organ antioxidant capacity and mitigate oxidative stress *in vivo*, showcasing their potential for combating oxidative damage

**Figure 5:**
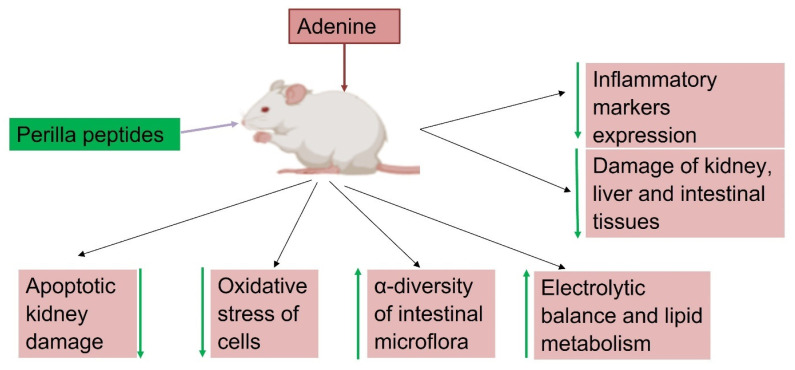
Perilla peptides mitigate adenine-induced kidney damage and delay the progression kidney injury through various mechanisms. (Figure created using Biorender.com).

Perilla peptides exhibit robust renal protective effects in adenine-induced renal injury models, addressing key aspects of kidney health including electrolyte balance, lipid metabolism, and osmolality regulation. Adenine administration disrupted electrolyte homeostasis, leading to significant deviations in serum levels of Ca^2+^, Mg^2+^, Cl^−^, Na^+^, and Pi, indicative of impaired kidney function. Treatment with perilla peptides effectively normalized these electrolyte imbalances, restoring Ca^2+^, Mg^2+^, Cl^−^, Na^+^, and Pi levels towards control values. Additionally, adenine exposure resulted in impaired kidney concentrating function, evident from reduced urinary osmolality and elevated serum osmolality. Treatment with perilla peptides restored urinary osmolality while decreasing serum osmolality, indicating enhanced renal tubular function and systemic osmotic balance regulation. Adenine treatment induced severe apoptotic damage in the kidneys of mice, as evidenced by reduced anti-apoptotic BCL2 expression and increased pro-apoptotic BAX and Caspase3 expressions. In contrast, high-dose perilla peptide intervention significantly enhanced BCL2 expression and decreased BAX and Caspase3 expressions, indicating the peptides’ potential in alleviating apoptotic kidney injury caused by adenine. Moreover, adenine-induced renal injury was associated with elevated serum levels of total cholesterol (T-CHO) and triglycerides (TG), characteristic of hyperlipidemia observed in chronic kidney disease (CKD) models. Perilla peptide intervention successfully reversed the heightened T-CHO and TG levels, highlighting their role in improving lipid metabolism and mitigating cardiovascular risk factors associated with kidney dysfunction [31]. These effects underscore the therapeutic potential of perilla peptides in mitigating renal damage and improving kidney function, emphasizing their multifaceted benefits in renal and systemic health management. Figure 5 illustrates the protective effects of perilla peptide against adenine-induced damage in mice, highlighting its role in delaying the progression of kidney damage. Perilla peptides reduce the expression of inflammatory markers, oxidative stress, and apoptotic kidney damage. They also improve histopathological outcomes. Additionally, these peptides enhance the expression of immunostimulatory cytokines, increase the α-diversity of intestinal microflora, maintain electrolytic balance, support lipid metabolism, and boost levels of antioxidant enzymes and proteins.

### 2.9. Perilla Peptides: Mitigating Chemical-Induced Sexual Dysfunction and Endocrine Disturbances

Cytochrome P450 side-chain cleavage enzyme (P450scc) is a mitochondrial enzyme involved in the conversion of cholesterol to pregnenolone, which is the first and rate-limiting step in the biosynthesis of all steroid hormones. This enzyme catalyzes the cleavage of the side-chain of cholesterol to form pregnenolone and is essential for steroidogenesis [84]. 3β-hydroxysteroid dehydrogenase (3β-HSD) is an enzyme that catalyzes the conversion of pregnenolone to progesterone, a key step in the biosynthesis of progestins and androgens. It plays a crucial role in the production of various steroid hormones by mediating the reduction of the 3-keto group of pregnenolone [85]. Steroidogenic acute regulatory protein (StAR) is a protein that facilitates the transport of cholesterol from the outer to the inner mitochondrial membrane, where P450scc is located. This transport of cholesterol is a rate-limiting step in steroid hormone biosynthesis, and StAR plays a pivotal role in regulating this process [86]. Steroidogenic factor-1 (SF-1) is a transcription factor that regulates the expression of genes encoding steroidogenic enzymes, including P450scc, 3β-HSD, and StAR. It plays a central role in the transcriptional regulation of genes involved in steroidogenesis, thereby controlling the synthesis of steroid hormones [87]. Hence, P450scc, 3β-HSD, StAR, and SF-1 are key components and regulators of the steroid hormone biosynthesis pathway, playing critical roles in the conversion of cholesterol to pregnenolone and subsequent synthesis of various steroid hormones.

**Figure 6:**
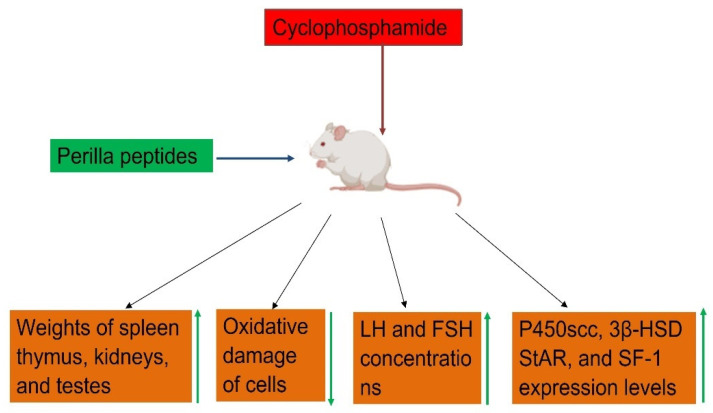
Schematic representation of perilla peptides mitigating cyclophosphamide induced sexual dysfunction and endocrine disturbances. Figure created using Biorender.com.

Cyclophosphamide-induced sexually impaired rats showed significant reductions in mRNA expression levels of P450scc, 3β-HSD, StAR, and SF-1, which play a critical role in the synthesis of sexual hormones. The coordinated regulation of these factors is essential for maintaining proper steroid hormone levels and physiological functions. Upon administration with perilla peptides, mRNA expression levels P450scc, 3β-HSD, StAR, and SF-1enzymes were significantly increased (Figure 6). Thus, perilla peptides showed protective function in rats from the sexual dysfunction caused by cyclophosphamide administration [83].

In rats subjected to cyclophosphamide-induced gonadotoxicity, a significant reduction in serum androgen levels, as well as a decrease in luteinizing hormone (LH) and follicle-stimulating hormone (FSH) concentrations, have been observed, indicative of disrupted endocrine function and impaired reproductive health. Cyclophosphamide, a potent chemotherapeutic agent, is known to induce these disturbances, affecting the biosynthesis and regulation of steroid hormones and reproductive hormones. However, a promising therapeutic intervention has emerged with the administration of perilla peptides. Supplementation with perilla peptides has demonstrated remarkable efficacy in restoring hormonal balance, as evidenced by a significant elevation in serum androgen levels and the restoration of LH and FSH to physiological concentrations (Figure 6). This indicates the potential of perilla peptides as a beneficial therapeutic strategy for preserving reproductive function and hormone homeostasis [83]. The observed restoration of hormonal balance underscores the significance of exploring natural peptide-based therapies in mitigating the endocrine disturbances associated with chemotherapy and improving overall reproductive health and quality of life. Further antioxidant activity and reverse of organ atrophy by perilla peptides mitigating the sexual dysfunction in rats injured with cyclophosphamide (Figure 6). This highlights the potential of perilla peptides as a promising therapeutic intervention to counteract the adverse effects of chemotherapy on reproductive endocrine function.

### 2.10. Anticancer Effects of Perilla Peptides

He et al., (2018) [25] identified and isolated three oligopeptide fractions, named perilla seed oligopeptides (PSO1, PSO2, and PSO3), from alcalase enzyme hydrolysate of PSM protein. They assessed the anticancer potential of these fractions against various human cancer cell lines, including lung adenocarcinoma (A549), colorectal cancer (HCT116), hepatic carcinoma (HepG2), human glioma cancer (U251), and human gastric carcinoma (MGC803). The study demonstrated that all PSOs exhibited significant cytotoxic effects across all tested cell lines. Particularly, PSO3 exhibited the most potent antiproliferative activity against U251 glioma cells, possibly related to its potential neurological benefits. Traditionally, perilla seeds have been used to treat neurological diseases, which may further support this finding. PSO1 and PSO2 also displayed notable anticancer effects against glioma cells, with PSO3 showing the highest efficacy, followed by PSO2 and then PSO1. In terms of A549, PSO3 was the most effective, followed by PSO1 and PSO2. Both PSO1 and PSO2 showed substantial anticancer activity against A549 as well. For HCT116 and HepG2, PSO3 again demonstrated the most significant antiproliferative effects, followed by PSO1 and PSO2. Moreover, PSO1 showed the strongest activity against MGC-803, followed by PSO3 and then PSO2. The anticancer efficacy ranking of the PSOs across the various cell lines is summarized as follows: PSO3 exhibited strong anticancer activity against U251, A549, and HCT116. However, it showed only moderate activity against HepG2 and MGC-803. PSO1 demonstrated strong anticancer effects against MGC-803, followed by U251 and HCT116. Its activity against A549 and HepG2 was comparatively weaker. PSO2 displayed the most potent anticancer activity against U251 but showed weaker activity against the remaining cell lines, including A549, HCT116, HepG2, and MGC-803. The amino acid sequence of PSO3 was confirmed as Ser-Gly-Pro-Val-Gly-Leu-Trp, while the sequences for PSO1 and PSO2 remain uncharacterized. The sequence of PSO3 provides insights into its potential anticancer mechanisms [25]. The specific amino acid composition, particularly the presence of proline (Pro) and tryptophan (Trp), may contribute to its enhanced anticancer activity. Proline residue can induce structural constraints, potentially leading to a more stable peptide conformation. This stability could enhance the peptide’s binding affinity to specific cellular targets, such as receptors or enzymes involved in cancer cell proliferation and survival. Tryptophan is known for its ability to interact with cell membranes and may facilitate the internalization of the peptide into cancer cells. Once inside the cell, the peptide could exert its cytotoxic effects, possibly by disrupting cellular processes essential for cancer cell survival [88,89]. Additionally, the presence of serine (Ser), glycine (Gly), and valine (Val) in the sequence might also contribute to the peptide’s biological activity by influencing its solubility, stability, and interactions with target molecules [90]. However, the exact molecular mechanisms underlying the anticancer effects of PSO3 would require further investigation, including studies on its specific cellular targets and signaling pathways. Further research would be necessary to fully elucidate the molecular mechanisms responsible for the observed anticancer activities of the PSO peptides.

### 2.11. Anti-Fatigue Effects of Perilla Seed Peptide

In a study by Liu et al., (2020) [30], perilla seed peptide was derived from perilla seed protein by treating it with neutral protease and papain enzymes. This perilla seed protein is composed of 18 amino acids, namely, Asp (aspartic acid), Thr (threonine), Ser (serine), Glu (glutamate), Gly (glycine), Ala (alanine), Val (valine), Cys (cysteine), Met (methionine), Ile (isoleucine), Leu (leucine), Tyr (tyrosine), Phe (phenylalanine), His (histidine), Lys (lysine), Arg (arginine), and Pro (proline). The impact of this perilla seed peptide on the exercise performance of mice was assessed through the exhaustive swimming test. In comparison to the control group, which swam for an average duration of 75.1 min, the mice supplemented with perilla seed peptide demonstrated a markedly increased swimming endurance, reaching 116.3 min. Furthermore, a notable enhancement in muscle strength was observed with perilla seed peptide supplementation. The average limb hanging time, initially around 61 s in the control group, extended to over 100 s after 4 weeks of perilla seed peptide administration. The physiological responses of mice during exercise were examined, with a focus on changes in serum LDH, BLA, BUN, and serum creatinine levels. Compared to the control group, administration of perilla seed peptide significantly elevated LDH levels, leading to a reduction in BLA. Perilla seed peptide supplementation effectively prevented the increase in BUN and CRE levels in mouse serum. Moreover, mice receiving perilla seed peptide showed a notable increase in liver glycogen (LG) and muscle glycogen (MG) levels compared to the control group. Additionally, perilla seed peptide efficiently reduced the fat coefficient and enhanced the muscle coefficient in the mice. Total serum protein (TP) levels were significantly elevated in mice fed perilla seed peptide. Muscle synthesis is governed by myogenic regulators, such as myogenin (MyoG) and myogenic differentiation factor MyoD. The levels of MyoG, MyoD, and TP were markedly increased in mice supplemented with perilla seed peptide. The study underscores the beneficial effects of perilla seed peptide supplementation on various physiological parameters in mice during exercise [30]. These effects include enhanced muscle synthesis, as indicated by increased levels of MyoG and MyoD, and improved metabolic profiles, including reduced levels of BLA, BUN, and CRE, and increased levels of LG, MG, and TP. These findings suggest that perilla seed peptide may have potential applications in enhancing exercise performance and muscle metabolism. Its ability to significantly improve exercise endurance and muscle strength in mice suggests its promising application in enhancing physical performance and muscle function.

In a study by Wei Ying (2021), perilla seed peptides exhibited the anti-fatigue effects as evaluated by measuring weight-loaded swimming time and analyzing muscle and liver glycogen levels, as well as BLA, BUN, and LDH activity. The results indicated a significant increase in the swimming time of mice across all dosage groups compared to the control group, demonstrating a dose-dependent enhancement in exercise capacity [91]. This suggests that perilla seed peptides can effectively improve physical endurance. The mechanism underlying the anti-fatigue activity of these peptides appears to involve multiple pathways. During exercise, muscle glycogen is primarily consumed, followed by liver glycogen, both of which are crucial energy sources. The study observed a substantial increase in both muscle and liver glycogen content in mice administered with high-dose perilla seed peptides, indicating enhanced glycogen accumulation and storage. This increase in glycogen stores provides additional energy, which is essential for prolonged physical activity and delaying the onset of fatigue. In situations where the body’s oxygen supply is insufficient, muscles switch from aerobic to anaerobic metabolism, leading to lactic acid buildup. Elevated lactic acid levels can impair the function of the circulatory and skeletal muscle systems, reducing exercise capacity. LDH plays a critical role in converting excess lactic acid into pyruvate, thus minimizing lactic acid accumulation and delaying fatigue. The study found that perilla seed peptides significantly increased LDH activity, facilitating more efficient lactic acid metabolism. Furthermore, the peptides accelerated the breakdown of BLA and BUN during exercise. This indicates an enhanced metabolic rate, which helps in the rapid clearance of these fatigue-related metabolites. The ability of perilla seed peptides to boost LDH activity and expedite the catabolism of BLA and BUN suggests that they support improved muscle recovery and sustained exercise performance.

The anti-fatigue effects of perilla seed peptides can be attributed to their multifaceted role in energy metabolism and muscle function. By increasing glycogen stores, enhancing LDH activity, and promoting the clearance of metabolic byproducts, these peptides provide a robust mechanism to combat fatigue and improve physical endurance. These findings suggest that perilla seed peptides could be developed into functional foods or supplements aimed at enhancing physical performance and mitigating fatigue. Further research into their mechanisms and potential applications could pave the way for new interventions in sports nutrition and fatigue management.

### 2.12. Perilla Seed Peptide Nanoparticles for Targeted Drug Delivery

Perilla seed oligopeptides (PSO), purified and extracted from perilla seeds, have shown promising anticancer properties. This study focuses on the development of perilla seed peptide nanoparticles (NPs) for targeted drug delivery, leveraging the unique properties of chitosan and hyaluronic acid (HA) for enhanced efficacy. To create the nanoparticles, electronegative HA was covalently bound to electropositive chitosan via EDC (1-ethyl-3-(3-dimethylaminopropyl) carbodiimide) chemistry, forming a stable assembly capable of effective drug loading. The resultant hyaluronic acid/perilla seed oligopeptide/chitosan (HA/PSO/C) NPs demonstrated a uniform particle size distribution with a mean diameter of 216.7 ± 4.5 nm, with high uniformity and colloidal stability. Drug encapsulation efficiency (EE) and loading efficiency (LE) were 38.7 ± 4.5% and 24.3 ± 1.2%, respectively, indicating successful incorporation of PSO into the nanoparticles. The release profile of the NPs exhibited an initial burst release of 60.7 ± 1.3% within the first 8 h, followed by a sustained release over 36 h, reaching a plateau at 81.1 ± 2.3% after 48 h. This release behavior is attributed to the chitosan matrix, which offers a controlled and prolonged drug release, enhancing the potential for sustained therapeutic effects and reduced systemic toxicity. The anticancer efficacy of HA/PSO/C NPs was assessed against five cancer cell lines. The NPs demonstrated significantly stronger cytotoxic effects compared to free PSO, with the most pronounced inhibition observed in U251 and A549 cell lines. The IC_50_ values for HA/PSO/C NPs at 24 and 48 h were 0.39 mg/mL and 0.22 mg/mL, respectively, compared to 2.00 mg/mL and 0.49 mg/mL for free PSO [25]. This enhancement is attributed to the synergistic effects of HA and chitosan. HA binds specifically to CD44 receptors, which are overexpressed on tumor cells, facilitating targeted delivery and increased drug concentration in the tumor microenvironment [92]. Chitosan, acting as a scaffold, ensures sustained release and further augments the therapeutic effect [93]. The targeting capability of HA is primarily due to its affinity for CD44 receptors on tumor cells. CD44, a cell surface glycoprotein, plays a crucial role in cell adhesion, migration, and proliferation. By binding to these receptors, HA facilitates the accumulation of nanoparticles in the tumor vicinity, thereby enhancing the localized drug concentration and minimizing off-target effects [92,93,94]. This targeted approach not only improves the efficacy of the encapsulated drug but also reduces potential side effects, making HA/PSO/C NPs a promising platform for cancer therapy. This study successfully demonstrates the potential of perilla seed peptide nanoparticles for targeted drug delivery. By utilizing the biocompatible and biodegradable properties of chitosan and HA, the formulated HA/PSO/C NPs offer a controlled release mechanism and enhanced anticancer efficacy through active targeting. The use of perilla seed peptides, a byproduct of oil extraction, also highlights the value of sustainable and comprehensive utilization of natural resources. Further research is warranted to explore the full therapeutic potential of PSO as an anticancer agent. Table 1 provides an overview of bioactive peptides derived from Perilla seed meal and their biological properties.

**Figure 7:**
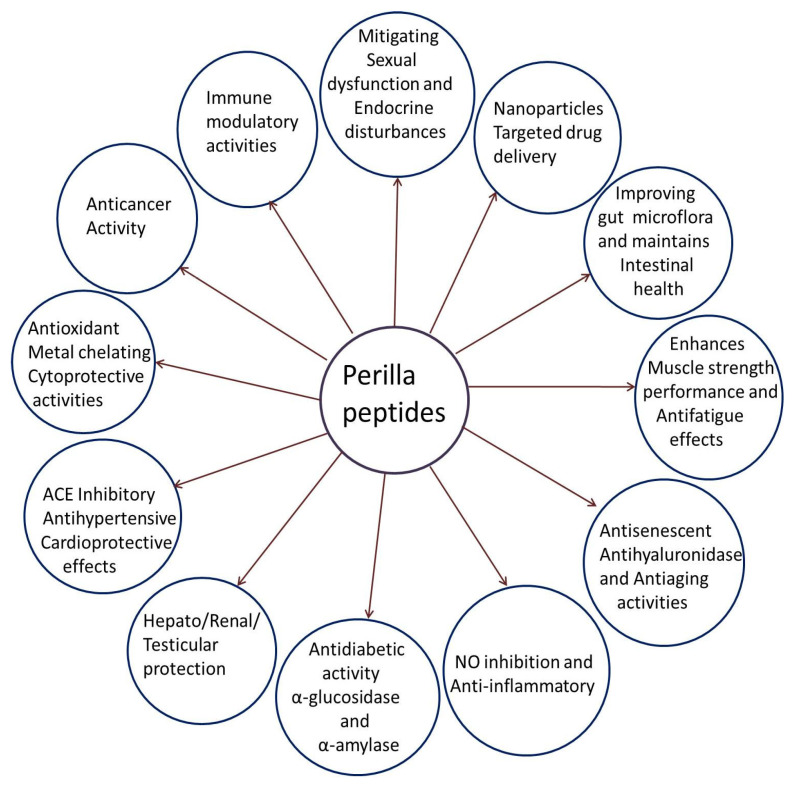
Schematic representation of multiple biological activities of perilla peptides.

In summary PSM protein hydrolysates and peptides exhibit a wide range of beneficial health effects, including metal chelating, antioxidant, cytoprotective, anti-aging, anti-inflammatory, antidiabetic, antihypertensive, anticancer, anti-fatigue, intestinal health maintenance, immunomodulatory, hepatoprotective, renal protective, and testicular protective properties. Further perilla seed oligopeptides have been employed for targeted drug delivery systems. Different biological activities of perilla peptides are represented in Figure 7.

## 3. Future Perspectives on Safety Evaluations of Perilla Proteins and Peptides

While the current review highlights the broad spectrum of beneficial bioactivities of perilla proteins and peptides, a critical gap exists in the literature regarding their safety and toxicological evaluations. Comprehensive safety studies are imperative to support their potential applications in food, pharmaceutical, and nutraceutical industries. Future research should prioritize *in vivo* acute and chronic toxicity assessments using established animal models, measuring key parameters such as mortality, behavioral changes, organ weights, serum biochemical markers (e.g., creatinine, ALP), and histopathological evaluations of vital organs. Such studies would provide foundational insights into the tolerability and safety thresholds of these bioactive compounds. Additionally, *in silico* safety evaluations should be undertaken to predict ADMET (absorption, distribution, metabolism, excretion, and toxicity) properties, bioavailability, and potential drug interactions. *In silico* approaches offer a cost-effective and efficient means to screen for potential toxicities, especially when designing functional food products or therapeutic agents.

Moreover, long-term studies addressing chronic toxicity, allergenicity, and genotoxicity are essential to establish the safety of perilla peptides for regular human consumption. Investigations into their effects on specific populations, such as those with pre-existing conditions or vulnerable groups like children and pregnant women, should also be conducted. These future perspectives will not only fill critical knowledge gaps but also enhance the confidence of consumers, regulators, and manufacturers in the safety and efficacy of perilla proteins and peptides as health-promoting agents. Such studies will serve as a cornerstone for advancing the therapeutic potential of perilla peptides while ensuring their safety for broader applications.

## 4. Conclusions

In this review, we highlight the diverse biological functions of perilla peptides, focusing on their extraction and purification steps, as well as the proteolytic enzymes and conditions used for these processes. Perilla peptides and protein hydrolysates demonstrate significant potential in combating oxidative stress and protecting cells from oxidative damage through mechanisms such as free radical scavenging, metal chelating, in vivo antioxidant, and cytoprotective activities. Perilla peptides exhibit robust anti-aging properties by activating the Nrf2 pathway, enhancing cellular antioxidant capacity, and supporting skin health by promoting keratinocyte growth, maintaining collagen integrity, and reducing senescent cells. Furthermore, perilla peptides show antidiabetic activity by inhibiting α-amylase and α-glucosidase. In terms of cardioprotective effects, perilla peptides inhibit ACE and combat oxidative stress through enhanced antioxidant defenses. They improve gut health by enhancing beneficial gut flora and reinforcing intestinal barriers. In liver, kidney, and testicular health, perilla peptides reduce oxidative stress and apoptotic damage while normalizing electrolyte levels. Additionally, they protect against cyclophosphamide-induced reproductive and endocrine disruptions by restoring hormone synthesis. Perilla peptides offer promising anticancer potential by inhibiting key cancer cell lines. They also exhibit anti-inflammatory and immunomodulating activities and demonstrate anti-fatigue effects, enhancing exercise performance and muscle function. Moreover, perilla seed peptide nanoparticles show potential for targeted drug delivery. These bioactivities are attributed to the specific amino acid compositions and low molecular weight of the peptides derived from perilla seeds, which are an important component in traditional Chinese cuisine and have been used for centuries in pickles, edible oils, and various food preparations. These diverse applications support the promising potential of perilla seed meal as functional food additives and therapeutic agents. Future directions include optimizing the processing, extraction, and purification of perilla peptides and further studying their biological mechanisms to facilitate their translation into clinical applications. Future directions for research on perilla peptides should prioritize comprehensive studies on their safety, tolerance, biocompatibility, and bioavailability. While numerous studies have highlighted their therapeutic and functional potential, a significant gap remains in understanding their toxicological profiles, long-term effects, and interactions within biological systems. Investigating these aspects will ensure the safe application of perilla peptides in clinical and industrial settings. Additionally, standardized protocols for assessing safety and efficacy, alongside advanced processing, extraction, and purification methods, are crucial. Such efforts will facilitate their seamless translation from laboratory studies to practical therapeutic and functional ingredient applications, ensuring both efficacy and safety for diverse populations. By advancing these areas, perilla can fulfil its potential as a multifaceted functional and therapeutic ingredient.

## Figures and Tables

**Table 1:** Overview of Bioactive Peptides Derived from Perilla Seed Meal: Sources, Characteristics, and Biological Activities.

S. No	Name of the Peptide(s)	Source of Perilla	Enzyme Used for Extraction	Peptide Sequence/A. A Composition	Mol. wt	Biological Activity	References
1.	PS-1	Perilla seed meal	Alkaline protease	Tyr-Leu (YL)	294.33 Da	Free radical scavenging activity Inhibition of lipid peroxidation Inhibition of hepatic cancer (HepG2 cells) Biocompatibility towards normal cells	Yang et al., 2018 [16].
2.	PS-2			Phe-Tyr (FY)	328.33 Da		
3.	Perilla peptides	Perilla seed meal	Acid protease Neutral protease Papain in the ratio of 2:4:1.5	Asp, Leu, Thr, Tyr, Ser, Phe, Glu, His, Gly, Lys, Ala, Arg, Val, Pro, Met, Cys and Ile	790 Da	Improved visceral organ indexes Ameliorated kidney and liver injury Improved kidney concentration function Improved antioxidant function capacity Electrolytic balance and lipid metabolism Amelioration of apoptotic lesions in kidneys Improved Intestinal microflora and gut health Suppression of inflammatory markers expression	Miangliang et al., 2021 [31].
4.	Perilla seed peptides	Perilla seed meal	Alkaline protease	Asp, Glu, Gly, Ala, Val, Leu, Ile, Ser, Thr, Phe, Tyr, His, Lys, Arg, Cys, Pro and Met	<1000 Da	Protective function in rats from sexual dysfunction by increasing mRNA expression levels of P450scc, 3β-HSD, StAR, and SF-1enzymes Immunomodulatory activity by increasing IL-2, IgG and IFN-ϒ Restoring hormonal balance and elevation of androgen levels	Miangliang et al., 2021 [31]
5.	PAP2	Perilla seed meal	Trypsin	Ile-Ser-Pro-Arg-Ile-Leu-Ser-Tyr-Asn-Leu-Arg	1330.77 Da	Free radical scavenging activity by DPPH, ABTS, and reducing power assays	Kim et al., 2019 [10]
6.	PAP1	Perilla seed meal	Trypsin	--------------------	-----------	Free radical scavenging activity by DPPH, ABTS, and reducing power assays	Kim et al., 2019 [10]
7.	PPSP (Purple perilla seed peptides with different mol wt)	Perilla seed meal	10:1 ratio of neutral protease/papain 10:1	Asp, Thr, Ser, Glu, Gly, Ala, Val, Cys, Met, Ile, Leu, Tyr, Phe, His, Lys, Arg, and Pro	150–1000 1000–2000 2000–3000 3000–5000 5000–10,000 >10,000	Antioxidant activity Upregulation of myogenic differentiation (MyoD) and myogenin (MyoG) Promotes muscles strength and exercise fatigue Increase visceral organ index of heart, liver, spleen, lungs, and kidneys	Liu et al., 2020 [30]
8.	Antioxidant peptide	Perilla seed meal	Alcalase	Lys-Leu-Lys-Asp-Ser-Phe-Glu-Arg-Gln-Gly-Met-Val	1437.8 Da	Metal chelating activity Inhibition of ROS via Fenton and Haber–Weiss reactions	Henghui et al., 2023 [22]
9.	PSO	Perilla seed sieved flour	Alcalase	Ser-Gly-Pro-Val-Gly-Leu-Trp	715.33 Da	Broad-spectrum anticancer activity with active targeting drug delivery Anticancer effects against cerebral glioma (U251), Lung carcinoma (A549), hepatic carcinoma (HepG2), Colon cancer (HCT116) and gastric adenocarcinoma (MGC-803) Targeted drug delivery	He et al., 2018 [25]
10.	PSO1 and PSO2	Perilla seed flour	Alcalase	---------------------	<3 KDa	Anticancer effects against U251, A549, HepG2, HCT116, and MGC-803	He et al., 2018 [25]
11.	Perilla peptides	Perilla Seed Meal	Flavourzyme		<1 kDa 1–3 kDa 3–5 kDa 5–10 kDa >10 kDa	Metal chelating activity Protection against lipid oxidative damage Enzyme-inhibitory activities such as α-amylase α-glucosidase and ACE-inhibitory Antidiabetic activity Antihypertensive activity	Park and Yoon, 2019 [14,20]
12.	Perilla peptides	Perilla Seed meal	Flavourzyme		<1 kDa 1–3 kDa 3–5 kDa 5–10 kDa >10 kDa	Radical scavenging activity by DPPH, ABTS, and reducing power assay Superoxide dismutase activity Functional food ingredients	Park and Yoon, 2018 [14,20]
13.	Perilla peptides	Perilla seed meal	Alcalase Neutrase Trypsin Papain Pepsin	Asp, Thr, Ser, Glu, Pro, Gly, Ala, Cys, Val, Trp, Met, Ile, Tyr, Phe, His, Lys, and Arg	1–27 kDa	Antioxidant activity by scavenging DPPH and ABTS free radicals Anti-inflammatory effects by NO-inhibition assay Antihypertensive effects by ACE-inhibitory activities	Kim and Yoon 2020 [18,19,50]
14.	Perilla peptide fractions UF-1, UF-2, and UF-3	Perilla seed meal	Alkaline protease	----------------	-------------	Free radical scavenging activity by inhibition of DPPH and ABTS free radicals	Hongyu et al., 2023 [48]
15.	UF-4 (fraction contains 18 potential bioactive peptides)	Perilla seed meal	Alkaline protease	CFFYR, HFFWN, ACVFAFM, WFHYH, ILGFYW, WILPFVP, GALFL, HIFFH, GGGFRG, GLGAFL, FITFR, KAFFY, FIASFL, RIWRP, LAVFW, FLFNK, WVTFY, GLFRI	<1 kDa	Protects cells from oxidative stress-induced damage by decreasing levels of MDA, increasing intracellular levels of GSH, enhancing the activity of antioxidant enzymes such as SOD and CAT, and reducing the activity of extracellular LDH. Modulates pathways involved in cell death and survival and protects against apoptosis in response to oxidative stress. Modulate inflammatory pathways to reduce oxidative stress-induced inflammation and inhibit key inflammatory signals such as TNF and IL-17 pathways. Engage in significant interactions with core proteins like SRC, STAT3, HSP90AA1, LCK, MAPK1, and FYN and influence multiple cellular processes through these interactions	Hongyu et al., 2023 [48]
16.	PSPs (NFF PMR)	Perilla seed meal	Alkaline protease	---------------------	3–5 KDa	Protection against oxidative damage Anti-senescent/anti-aging activity Promote the proliferation of keratinocytes and inhibits the level of ROS and the activity of β- galactosidase Downregulated the expression of matrix metalloproteinases (MMPs) and the degradation of collagens (COLs) Competitive binding of Keap1 to facilitate the release of Nrf2 and activation of NF-κB signal pathway	Wang et al., 2024 [24].

## Data Availability

No new data were created or analyzed in this study. Data sharing is not applicable to this article.

## References

[1] Ahmed H.M. (2018). Ethnomedicinal, phytochemical and pharmacological investigations of *Perilla frutescens* (L.) Britt. Molecules.

[2] Wu X., Dong S., Chen H., Guo M., Sun Z., Luo H. (2023). Perilla frutescens: A traditional medicine and food homologous plant. Chin. Herb. Med..

[3] Dhyani A., Chopra R., Garg M. (2019). A review on nutritional value, functional properties and pharmacological application of perilla (*Perilla frutescens* L.). Biomed. Pharmacol. J..

[4] Dossou S.S.K., Deng Q., Li F., Jiang N., Zhou R., Wang L., Li D., Tan M., You J., Wang L. (2023). Comparative Metabolomics Analysis of Different Perilla Varieties Provides Insights into Variation in Seed Metabolite Profiles and Antioxidant Activities. Foods.

[5] Kaur S., Seem K., Ali A., Jaiswal S., Gumachanamardi P., Kaur G., Singh N., Touthang L., Kumar S., Bhardwaj R. (2024). A comprehensive review on nutritional, nutraceutical, and industrial perspectives of perilla (*Perilla frutscens* L.) seeds–An orphan oilseed crop. Heliyon.

[6] Hou T., Netala V.R., Zhang H., Xing Y., Li H., Zhang Z. (2022). *Perilla frutescens*: A rich source of pharmacological active compounds. Molecules.

[7] Longvah T., Deosthale Y.G. (1991). Chemical and nutritional studies on Hanshi (*Perilla frutescens*), a traditional oilseed from Northeast India. J. Am. Oil Chem. Soc..

[8] Bondioli P., Folegatti L., Rovellini P. (2020). Oils rich in alpha linolenic acid: Chemical composition of perilla (*Perilla frutescens*) seed oil. OCL.

[9] Tantipaiboonwong P., Chaiwangyen W., Suttajit M., Kangwan N., Kaowinn S., Khanaree C., Punfa W., Pintha K. (2021). Molecular Mechanism of Antioxidant and Anti-Inflammatory Effects of Omega-3 Fatty Acids in Perilla Seed Oil and Rosmarinic Acid Rich Fraction Extracted from Perilla Seed Meal on TNF-α Induced A549 Lung Adenocarcinoma Cells. Molecules.

[10] Kim J.M., Liceaga A.M., Yoon K.Y. (2019). Purification and identification of an antioxidant peptide from perilla seed (*Perilla frutescens*) meal protein hydrolysate. Food Sci. Nutr..

[11] Parry J., Yu L. (2004). Fatty acid content and antioxidant properties of cold-pressed black raspberry seed oil and meal. J. Food Sci..

[12] Pintathong P., Chaiwut P., Thitipramote N., Thitilertdecha N., Nantitanont W., Sangthong S., Tiensri N. (2018). Simultaneous extraction of oil and protein from perilla seed by three-phase partitioning and their application in serum. J. Appsci..

[13] Song N.B., Lee J.H., Song K.B. (2015). Preparation of perilla seed meal protein composite films containing various essential oils and their application in sausage packaging. J. Korean Soc. Appl. Biol. Chem..

[14] Park B.Y., Yoon K.Y. (2019). Biological activity of enzymatic hydrolysates and the membrane ultrafiltration fractions from perilla seed meal protein. Czech J. Food Sci..

[15] Zhang H., Zhang Z., He D., Li S., Xu Y. (2022). Optimization of enzymatic hydrolysis of Perilla meal protein for hydrolysate with high hydrolysis degree and antioxidant activity. Molecules.

[16] Yang J., Hu L., Cai T., Chen Q., Ma Q., Yang J., Meng C., Hong J. (2018). Purification and identification of two novel antioxidant peptides from perilla (*Perilla frutescens* L. Britton) seed protein hydrolysates. PLoS ONE.

[17] Wang D., Li H., Hou T.Y., Zhang Z.J., Li H.Z. (2024). Effects of conjugated interactions between Perilla seed meal proteins and different polyphenols on the structural and functional properties of proteins. Food Chem..

[18] Kim J.M., Yoon K.Y. (2021). Effects of pH, heat, and intestinal protease treatments on antioxidant activity of antioxidant peptides derived from protein hydrolysate of perilla seed meal. Food Sci. Preserv..

[19] Kim J.M., Yoon K.Y. (2020). Determination of protein extraction and trypsin hydrolysis conditions for producing hydrolysates with antioxidant activity from perilla seed meal. Food Sci. Preserv..

[20] Park B.Y., Yoon K.Y. (2019). Functional properties of enzymatic hydrolysate and peptide fractions from perilla seed meal protein. Polish J. Food Nutr. Sci..

[21] Kamboj A., Chopra R., Singh R., Saxena V., GV P.K. (2022). Effect of pulsed electric field parameters on the alkaline extraction of valuable compounds from perilla seed meal and optimization by central composite design approach. Appl. Food Res..

[22] Henghui Z., Zhang Z., Chen S., Ye X., Zhang G. (2023). Preparation, isolation, purification and sequence identification of antioxidant peptide from Perilla meal protein. J. Chin. Inst. Food Sci. Tech..

[23] Zhao Q., Wang L., Hong X., Liu Y., Li J. (2021). Structural and functional properties of perilla protein isolate extracted from oilseed residues and its utilization in Pickering emulsions. Food Hydrocolloids..

[24] Wang L., Qu L., He B. (2024). Preparation, Identification and Molecular Docking of two Novel Anti-aging peptides from Perilla Seed. Heliyon.

[25] He D.L., Jin R.Y., Li H.Z., Liu Q.Y., Zhang Z.J. (2018). Identification of a novel anticancer oligopeptide from *Perilla frutescens* (L.) britt.; its enhanced anticancer effect by targeted nanoparticles in vitro. Int. J. Polym. Sci..

[26] Jeong K., Lee S.Y., Jeon S.A., Gantulga P., Nam J.Y., Hong S.J., Lee S. (2021). Clinical and Immunological Characterization of Perilla Seed Allergy in Children. J. Investig. Allergol. Clin. Immunol..

[27] Oita S., Kimura T., Shibuya Y., Nihei N., Kon T. (2008). Extraction and digestibility of perilla frutescens seed proteins. Jpn. Agric. Res..

[28] Hao L., Lv C., Cui X., Yi F., Su C. (2021). Study on biological activity of perilla seed oil extracted by supercritical carbon dioxide. LWT.

[29] Zhou X.J., Yan L.L., Yin P.P., Shi L.L., Zhang J.H., Liu Y.J., Ma C. (2014). Structural characterisation and antioxidant activity evaluation of phenolic compounds from cold-pressed *Perilla frutescens* var. arguta seed flour. Food Chem..

[30] Liu Y., Li D., Wei Y., Ma Y., Wang Y., Huang L., Wang Y. (2020). Hydrolyzed peptides from purple perilla (*Perilla frutescens* L. Britt) seeds improve muscle synthesis and exercise performance in mice. J. Food Biochem..

[31] Miangliang L., Wei Y., Cai M., Gu R., Pan X., Du J. (2021). Perilla peptides delay the progression of kidney disease by improving kidney apoptotic injury and oxidative stress and maintaining intestinal barrier function. Food Biosci..

[32] Wen C., Zhang J., Zhang H., Duan Y., Ma H. (2020). Plant protein-derived antioxidant peptides: Isolation, identification, mechanism of action and application in food systems: A review. Trends Food Sci. Technol..

[33] Park B.Y., Yoon K.Y. (2018). Conditions for hydrolysis of perilla seed meal protein for producing hydrolysates and ultrafiltered peptides and their antioxidant activity. Food Sci. Preserv..

[34] Adjimani J.P., Asare P. (2015). Antioxidant and free radical scavenging activity of iron chelators. Toxicol. Rep..

[35] Chaudhary P., Janmeda P., Docea A.O., Yeskaliyeva B., Abdull Razis A.F., Modu B., Calina D., Sharifi-Rad J. (2023). Oxidative stress, free radicals and antioxidants: Potential crosstalk in the pathophysiology of human diseases. Front. Chem..

[36] Zou T.B., He T.P., Li H.B., Tang H.W., Xia E.Q. (2015). The Structure-Activity Relationship of the Antioxidant Peptides from Natural Proteins. Molecules.

[37] Ghribi A.M., Sila A., Przybylski R., Nedjar-Arroume N., Makhlouf I., Blecker C., Attia H., Dhulster H., Bougatef A., Besbes S. (2015). Purification and identification of novel antioxidant peptides from enzymatic hydrolysate of chickpea (*Cicer arietinum* L.) protein concentrate. J. Funct. Foods.

[38] Chi C., Wang B., Wang Y., Zhang B., Deng S. (2015). Isolation and characterization of three antioxidant peptides from protein hydrolysate of bluefin leatherjacket (*Navodon septentrionalis*) heads. J. Funct. Foods.

[39] Hsu K. (2010). Purification of antioxidative peptides prepared from enzymatic hydrolysates of tuna dark muscle by-product. Food Chem..

[40] Ranathunga S., Rajapakse N., Kim S. (2006). Purification and characterization of antioxidative peptide derived from muscle of conger eel (*Conger myriaster*). Eur. Food Res. Technol..

[41] Sarmadi B.H., Ismail A. (2010). Antioxidative peptides from food proteins: A review. Peptides.

[42] He R., Girgih A.T., Malomo S.A., Ju X., Aluko R.E. (2013). Antioxidant activities of enzymatic rapeseed protein hydrolysates and the membrane ultrafiltration fractions. J. Funct. Foods.

[43] Wang S.Y., Camp M.J., Ehlenfeldt M.K. (2012). Antioxidant capacity and α-glucosidase inhibitory activity in peel and flesh of blueberry (Vaccinium s) cultivars. Food Chem..

[44] Memarpoor-Yazdi M., Asoodeh A., Chamani J. (2012). A novel antioxidant and antimicrobial peptide from hen egg white lysozyme hydrolysates. J. Funct. Foods.

[45] Chen H., Zhao M., Lin L., Wang J., Sun-Waterhouse D., Dong Y., Zhuang M., Su G. (2015). Identification of antioxidative peptides from defatted walnut meal hydrolysate with potential for improving learning and memory. Food Res. Int..

[46] He R., Ju X., Yuan J., Wang L., Girgih A.T., Aluko R.E. (2012). Antioxidant activities of rapeseed peptides produced by solid state fermentation. Food Res. Int..

[47] Bougatef A., Nedjar-Arroume N., Manni L., Ravallec R., Barkia A., Guillochon D., Nasri M. (2010). Purification and identification of novel antioxidant peptides from enzymatic hydrolysates of sardinelle (*Sardinella aurita*) by-products proteins. Food Chem..

[48] Hongyu Z., Li H.Z., Zhijun Z., Yana Z., Hou T., Li H., Lin C. (2023). Exploration of mechanism of anti-oxidative stress of perilla meal peptides based on in vitro experiments and network pharmacology. Food Ferment. Ind..

[49] Chi C.F., Cao Z.H., Wang B., Hu F.Y., Li Z.R., Zhang B. (2014). Antioxidant and functional properties of collagen hydrolysates from Spanish mackerel skin as influenced by average molecular weight. Molecules.

[50] Kim J.M., Yoon K.Y. (2020). Functional properties and biological activities of perilla seed meal protein hydrolysates obtained by using different proteolytic enzymes. Food Sci. Biotechnol..

[51] Nguyen E., Jones O., Kim Y.H.B., San Martin-Gonzalez F., Liceaga A.M. (2017). Impact of microwave-assisted enzymatic hydrolysis on functional and antioxidant properties of rainbow trout Oncorhynchus mykiss by-products. Fish. Sci..

[52] Li J., Wang J., Zhang N., Li Y., Cai Z., Li G., Liu Z., Liu Z., Wang Y., Shao X. (2023). Anti-aging activity and their mechanisms of natural food-derived peptides: Current advancements. Food Innov. Adv..

[53] Ulasov A.V., Rosenkranz A.A., Georgiev G.P., Sobolev A.S. (2022). Nrf2/Keap1/ARE signaling: Towards specific regulation. Life Sci..

[54] Vomund S., Schäfer A., Parnham M.J., Brüne B., Von Knethen A. (2017). Nrf2, the master regulator of anti-oxidative responses. Int. J. Mol. Sci..

[55] Poovitha S., Parani M. (2016). In vitro and in vivo α-amylase and α-glucosidase inhibiting activities of the protein extracts from two varieties of bitter gourd (*Momordica charantia* L.). BMC Complement. Altern. Med..

[56] Gong L., Feng D., Wang T., Ren Y., Liu Y., Wang J. (2020). Inhibitors of α-amylase and α-glucosidase: Potential linkage for whole cereal foods on prevention of hyperglycemia. Food Sci. Nutr..

[57] Kang M.G., Yi S.H., Lee J.S. (2013). Production and characterization of a new α-glucosidase inhibitory peptide from Aspergillus oryzae N159-1. Mycobiology.

[58] PEACE Trial Investigators (2004). Angiotensin-converting–enzyme inhibition in stable coronary artery disease. N. Engl. J. Med..

[59] Xue L., Yin R., Howell K., Zhang P. (2021). Activity and bioavailability of food protein-derived angiotensin-I-converting enzyme–inhibitory peptides. Compr. Rev. Food Sci. Food Saf..

[60] Dong J., Wang S., Yin X., Fang M., Gong Z., Wu Y. (2022). Angiotensin I converting enzyme (ACE) inhibitory activity and antihypertensive effects of rice peptides. Food Sci. Hum. Wellness.

[61] Daskaya-Dikmen C., Yucetepe A., Karbancioglu-Guler F., Daskaya H., Ozcelik B. (2017). Angiotensin-I-converting enzyme (ACE)-inhibitory peptides from plants. Nutrients.

[62] Li Y., Pan D., Zhang W., Xie X., Dang Y., Gao X. (2024). Identification and molecular mechanism of novel ACE inhibitory peptides from broccoli protein. Food Biosci..

[63] Liu K., Gao Z., Li Q., Zhang H. (2023). Identification and mechanistic study of four novel ACE inhibitory peptides from maize germ protein hydrolysates. LWT.

[64] Griendling K.K., Camargo L.L., Rios F.J., Alves-Lopes R., Montezano A.C., Touyz R.M. (2021). Oxidative stress and hypertension. Circ. Res..

[65] Schulz E., Gori T., Münzel T. (2011). Oxidative stress and endothelial dysfunction in hypertension. Hypertens. Res..

[66] Cao W., Zhang C., Hong P., Ji H., Hao J. (2010). Purification and identification of an ACE inhibitory peptide from the peptic hydrolysate of Acetes chinensis and its antihypertensive effects in spontaneously hypertensive rats. Int. J. Food Sci. Technol..

[67] Xie J., Du M., Shen M., Wu T., Lin L. (2019). Physico-chemical properties, antioxidant activities and angiotensin-I converting enzyme inhibitory of protein hydrolysates from Mung bean (*Vigna radiate*). Food Chem..

[68] Picariello G., Ferranti P., Fierro O., Mamone G., Caira S., Di Luccia A., Monica S., Addeo F. (2010). Peptides surviving the simulated gastrointestinal digestion of milk proteins: Biological and toxicological implications. J. Chromatogr. B.

[69] De Vos W.M., Tilg H., Van Hul M., Cani P.D. (2022). Gut microbiome and health: Mechanistic insights. Gut.

[70] Fan Y., Pedersen O. (2021). Gut microbiota in human metabolic health and disease. Nat. Rev. Microbiol..

[71] Thursby E., Juge N. (2017). Introduction to the human gut microbiota. Biochem. J..

[72] Wu I.W., Lin C.Y., Chang L.C., Lee C.C., Chiu C.Y., Hsu H.J., Sun C.Y., Chen Y.C., Kuo Y.L., Yang C.W. (2020). Gut microbiota as diagnostic tools for mirroring disease progression and circulating nephrotoxin levels in chronic kidney disease: Discovery and validation study. Int. J. Biol. Sci..

[73] Zhao J., Ning X., Liu B., Dong R., Bai M., Sun S. (2021). Specific alterations in gut microbiota in patients with chronic kidney disease: An updated systematic review. Ren. Fail..

[74] Kuo W.T., Odenwald M.A., Turner J.R., Zuo L. (2022). Tight junction proteins occludin and ZO-1 as regulators of epithelial proliferation and survival. Ann. N. Y. Acad. Sci..

[75] Wang B., Wu G., Zhou Z., Dai Z., Sun Y., Ji Y., Li W., Wang W., Liu C., Han F. (2015). Glutamine and intestinal barrier function. Amino Acids.

[76] Ruth M.R., Field C.J. (2013). The immune modifying effects of amino acids on gut-associated lymphoid tissue. J. Anim. Sci. Biotechnol..

[77] Chow W.Y., Forman C.J., Bihan D., Puszkarska A.M., Rajan R., Reid D.G., Slatter D.A., Colwell L.J., Wales D.J., Farndale R.W. (2018). Proline provides site-specific flexibility for in vivo collagen. Sci. Rep..

[78] Li P., Wu G. (2018). Roles of dietary glycine, proline, and hydroxyproline in collagen synthesis and animal growth. Amino Acids.

[79] Sharma J.N., Al-Omran A., Parvathy S.S. (2007). Role of nitric oxide in inflammatory diseases. Inflammopharmacology.

[80] Ahn C., Cho Y., Je J. (2015). Purification and anti-inflammatory action of tripeptide from salmon pectoral fin byproduct protein hydrolysate. Food Chem..

[81] Rodríguez-Yoldi M.J. (2021). Anti-Inflammatory and Antioxidant Properties of Plant Extracts. Antioxidants.

[82] Rivera-Jiménez J., Berraquero-García C., Pérez-Gálvez R., García-Moreno P.J., Espejo-Carpio F.J., Guadix A., Guadix E.M. (2022). Peptides and protein hydrolysates exhibiting anti-inflammatory activity: Sources, structural features and modulation mechanisms. Food Funct..

[83] Miangliang L., Jiang S., Guo Y., Chen L., Wang Y.C., Cai M.Y., Gu R.Z., Wei Y. (2021). Ameliorative effect of perilla seed peptide on cyclophosphamide-induced sexual function impairment of rats. Food Sci..

[84] Li N., Oakes J.A., Storbeck K.H., Cunliffe V.T., Krone N.P. (2020). The P450 side-chain cleavage enzyme Cyp11a2 facilitates steroidogenesis in zebrafish. J. Endocrinol..

[85] Rasmussen M.K., Ekstrand B., Zamaratskaia G. (2013). Regulation of 3β-hydroxysteroid dehydrogenase/Δ⁵-Δ⁴ isomerase: A review. Int. J. Mol. Sci..

[86] Tugaeva K.V., Sluchanko N.N. (2019). Steroidogenic Acute Regulatory Protein: Structure, Functioning, and Regulation. Biochemistry.

[87] Relav L., Doghman-Bouguerra M., Ruggiero C., Muzzi J.C.D., Figueiredo B.C., Lalli E. (2023). Steroidogenic Factor 1, a Goldilocks Transcription Factor from Adrenocortical Organogenesis to Malignancy. Int. J. Mol. Sci..

[88] Patriarca E.J., Cermola F., D’Aniello C., Fico A., Guardiola O., De Cesare D., Minchiotti G. (2021). The Multifaceted Roles of Proline in Cell Behavior. Front. Cell Dev. Biol..

[89] Palego L., Betti L., Rossi A., Giannaccini G. (2016). Tryptophan Biochemistry: Structural, Nutritional, Metabolic, and Medical Aspects in Humans. J. Amino Acids..

[90] Ceballos-Alcantarilla E., Merkx M. (2021). Understanding and applications of Ser/Gly linkers in protein engineering. Methods in Enzymology.

[91] Ying W., Ying G., Li M., Liang C., Wang C., Sheng J., Gu R. (2021). Anti-fatigue Activity and Mechanisms of Peptides Derived from Perilla frutescens Seeds. J. Chin. Inst. Food Sci. Tech..

[92] Luo Z., Dai Y., Gao H. (2019). Development and application of hyaluronic acid in tumor targeting drug delivery. Acta Pharm. Sin. B.

[93] Gholap A.D., Rojekar S., Kapare H.S., Vishwakarma N., Raikwar S., Garkal A., Mehta T.A., Jadhav H., Prajapati M.K., Annapure U. (2024). Chitosan scaffolds: Expanding horizons in biomedical applications. Carbohydr. Polym..

[94] Senbanjo L.T., Chellaiah M.A. (2017). CD44: A Multifunctional Cell Surface Adhesion Receptor Is a Regulator of Progression and Metastasis of Cancer Cells. Front. Cell Dev. Biol..

